# What it takes to reduce sitting at work: a pilot study on the effectiveness and correlates of a multicomponent intervention

**DOI:** 10.1007/s00420-023-02020-4

**Published:** 2023-11-10

**Authors:** Jannik Porath, Laura I. Schmidt, Juliane Möckel, Chiara Dold, Lisa Hennerkes, Alexander Haussmann

**Affiliations:** 1 Institute of Psychology, Heidelberg University, Heidelberg, Germany; 2 Department for Prevention and Health Promotion, Heidelberg University of Education, Heidelberg, Germany; 3 Division of Physical Activity, Prevention and Cancer, German Cancer Research Center (DFKZ), Heidelberg, Germany

**Keywords:** Sedentary behavior, Health behavior change, Workplace intervention, Multicomponent intervention, Health action process approach, Occupational health

## Abstract

**Objective:**

This study aimed to assess the feasibility and effects of a simple-to-implement multicomponent intervention to reduce sedentary time of office workers.

**Methods:**

Six groups of eight to ten office workers took part in the two-week *Leicht Bewegt* intervention. Participants completed questionnaires at baseline (T0, *n* = 52), after 2 weeks (T1, *n* = 46), and after 5 weeks (T2, *n* = 38), including subjective sedentary measures and social-cognitive variables based on the health action process approach (HAPA). Objective sedentary measures were obtained using *activPAL* trackers.

**Results:**

The intention to reduce sedentary behavior during work increased significantly from T0 to T1. Participants’ objective and subjective sitting time decreased significantly from T0 to T1, corresponding to an average decrease per 8-h-workday of 55 min (*d* = − .66) or 74 min (*d* = − 1.14), respectively. This reduction persisted (for subjective sitting time) at T2 (*d* = − 1.08). Participants indicated a high satisfaction with the intervention.

**Conclusions:**

The *Leicht Bewegt* intervention offers a feasible and effective opportunity to reduce sedentary behavior at work. Randomized controlled trials including longer follow-up time periods are needed to validate its benefits in different workplaces.

**Supplementary Information:**

The online version contains supplementary material available at 10.1007/s00420-023-02020-4.

## Introduction

Sitting is the new smoking—although a disputed slogan (Vallance et al. 2018)—current research gives strong indications that prolonged sedentary time is indeed associated with a significant increase in the relative risk of numerous negative health outcomes, namely the incidence of cardiovascular diseases, cancer, type 2 diabetes, orthopedic problems, and all-cause mortality (Biswas et al. 2015; Kang et al. 2020; Park et al. 2020; Patterson et al. 2018). In light of these serious health threats, the World Health Organization (2020) recommends to limit the time spent sedentary and stresses health benefits of replacing it with physical activity of any intensity. The workplace belongs to the key settings of excessive sedentary times (Thorp et al. 2012). German office workers spend a median of 11 h a day sitting, 4 h longer than the total German population on a normal working day (Froböse and Wallmann-Sperlich 2016). Further studies indicate an average proportion of sedentary time in office jobs of approximately 80% of the working hours (Parry and Straker 2013; Rosenkranz et al. 2020). Accordingly, office workplaces represent a promising place to target unhealthy sitting habits.

### Previous intervention approaches to reduce sedentary behavior

In an umbrella review, Nguyen et al. (2020) found that sedentary behavior interventions in workplace-settings led to significant sedentary time reductions of at least 30 min per day. Backé et al. (2018) focused on these workplace-interventions and pointed out the common subdivision of intervention approaches in four different types: workplace environment interventions, individual-centered interventions, organizational interventions, and multi-component interventions that represent a combination of the other three types. Reviews indicate multi-component interventions to be the best approach to induce a sustainable change in sedentary behavior in the workplace (Backé et al. 2018; Chu et al. 2016). They are characterized by combining lasting modifications of processes and the working environment with a collective consciousness and information on how to achieve a healthy sedentary behavior.

Despite a remarkable number of intervention trials on sedentary behavior, a Cochrane review postulated low evidence of all types of workplace interventions to reduce sedentary behaviors mainly due to studies’ quality (Shrestha et al. 2018). A lack of standardized and objectively determined outcome measures (Chu et al. 2016) and an exclusive reliance on participants’ subjective reports (Prince et al. 2020) pose main problems. Cost-effectiveness analyses of multicomponent interventions to reduce sedentary times at workplace further show that, although they are likely to pay off in the long run from a health economics perspective, they are associated with substantial initial costs to the employer—particularly if height-adjustable workstations need to be purchased (Cox et al. 2022; Michaud et al. 2022).

### The Leicht Bewegt intervention

In the present study, we investigated the feasibility, the effectiveness as well as psychological mechanisms of the *Leicht Bewegt* intervention. Based on the validated Australian intervention programs *StandUp Australia* (Neuhaus et al. 2014a, b) and *BeUpstanding* (Healy et al. 2016), *Leicht Bewegt* represents a translated and culturally adapted German intervention toolkit. The intervention follows a group-based approach: Guided by an in-group motivator and coordinator (i.e., *active champion*), strategies are collaboratively discussed and selected that the group expects to have the greatest impact in reducing sitting time in their specific work environment. Selected strategies can address multiple components by targeting the physical environment, organizational processes, and/or individual behaviors. Since the intervention can be performed by employees themselves without the need for extra equipment, it offers the potential of a way to reduce sedentary behavior in the workplace that is not only effective, but also economical and easy to implement.

### The health action process approach (HAPA)

**Fig. 1:**
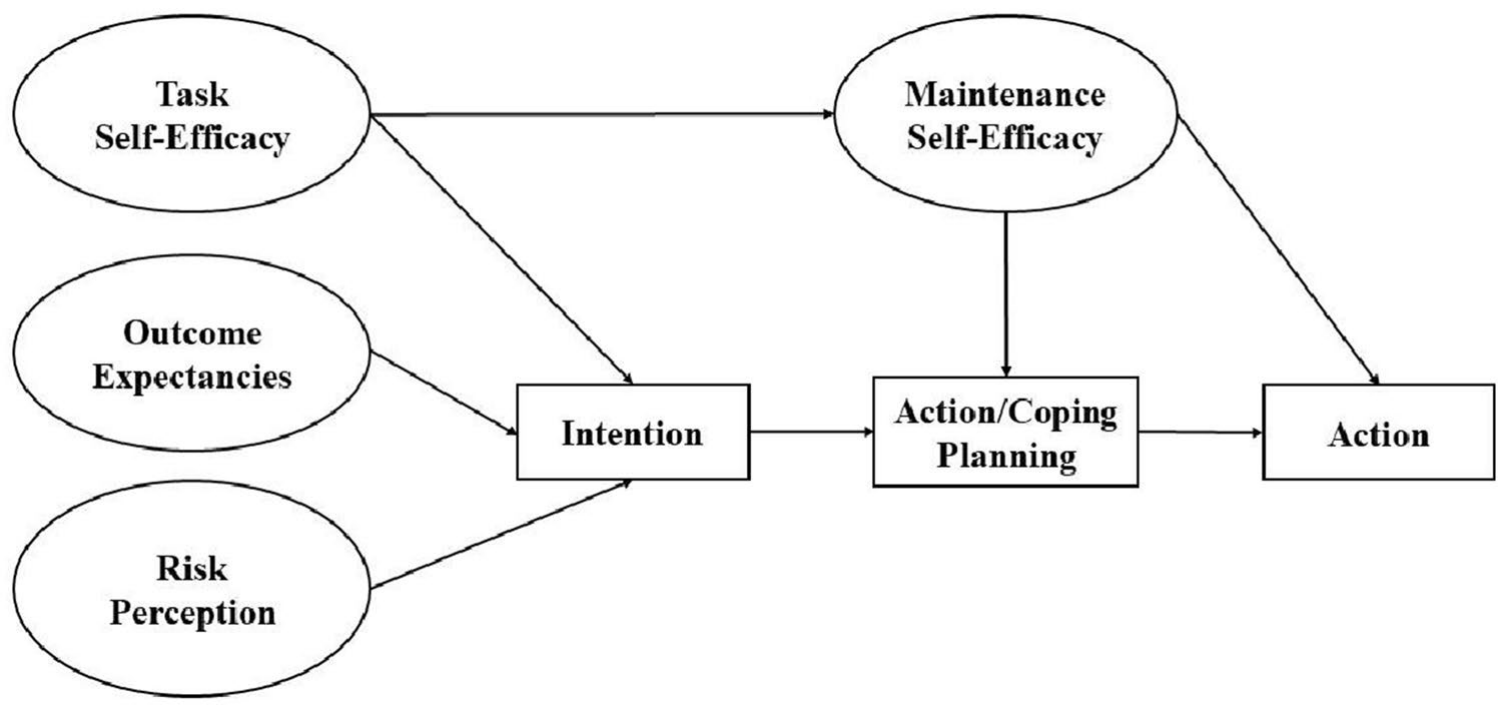
Health action process approach (Schwarzer 2008); version of the present study

Backé et al. (2018) considered the research on the psychological mechanisms of workplace interventions to reduce sedentary time to be incomplete. The *health action process approach* (HAPA; Schwarzer 2008) represents an established theoretical framework to illustrate health behavior change processes. In the motivational phase, the model postulates three antecedents of intention: *risk awareness*, *outcome expectancies*, and *task self-efficacy*. After the forming of an intention, the behavior change process enters the volitional phase with *action/coping planning* and *maintenance self-efficacy* as predictors of behavior. Figure 1 shows the HAPA model as used in the present study.

Previous studies on the HAPA framework provide indications for its general applicability to sedentary behavior (Maher and Conroy 2016; Rollo and Prapavessis 2020a, b; Zhang et al. 2019). However, multicomponent interventions have not been investigated drawing on the HAPA although this would allow a simultaneous consideration of motivational and volitional constructs for behavior change.

This pilot study aims to (a) explore the feasibility and acceptance of the *Leicht Bewegt* intervention, (b) analyze the effectiveness of the intervention on the objectively assessed and subjectively reported physical activity during working hours, and (c) explore potential psychological correlates of intervention mechanisms based on the HAPA model.

## Methods

### Recruitment and sample

The present study was conducted between May and July 2021 as a pilot project to introduce the *Leicht Bewegt* intervention for office workers at the German Cancer Research Center in Heidelberg (DKFZ), Germany. Ethical approval was obtained by the ethics commission of the Faculty of Behavioral and Cultural Studies of the University of Heidelberg (protocol number: AZ Schm 2021 1/1). The corporate health management of the DKFZ sent an email to all employees (around 3,200) with information on the study to recruit study participants. Study participation was voluntary and independent of any work-related benefits. Furthermore, participation in all study-related content such as workshops or questionnaires was allowed to occur within working hours. As inclusion criteria, we defined fluency in German, employment with the DKFZ, and a regular DKFZ-workplace in Heidelberg, Germany. Since the program is designed as a group intervention, we asked employees to enroll directly as a group of about ten individuals including one designated active champion. Based on the availability of 30 activity trackers and the results of our power analysis (see below), we decided to form two cohorts (each with 30 participants) that began the study only one week apart.

Initially, six groups with ten participants each registered. After receiving the first six complete group registrations, we ended the registration phase. Five individuals dropped out due to illness or (spontaneous) absence before the start of the study, so that the six groups consisted of eight to ten members. A total of *n* = 55 employees, working in the administration and research sections of the DKFZ, were finally registered in the study (83.6% female). Most participants indicated to be full-time workers (60%) and were between 46 and 65 years old (45.5%). A majority of participants (62.3%) reported to alternate between home office and in-office presence work.

The assignment to the two cohorts was intended to be completely at random. However, based on participants planned absences (that they had to indicate during registration), this would have led to a considerable number of data gaps. Therefore, we decided to perform cohort assignment manually to allow as many participants as possible to fully participate in the study. Informed consent was obtained from all participants included in the study.

### Procedure

**Fig. 2:**
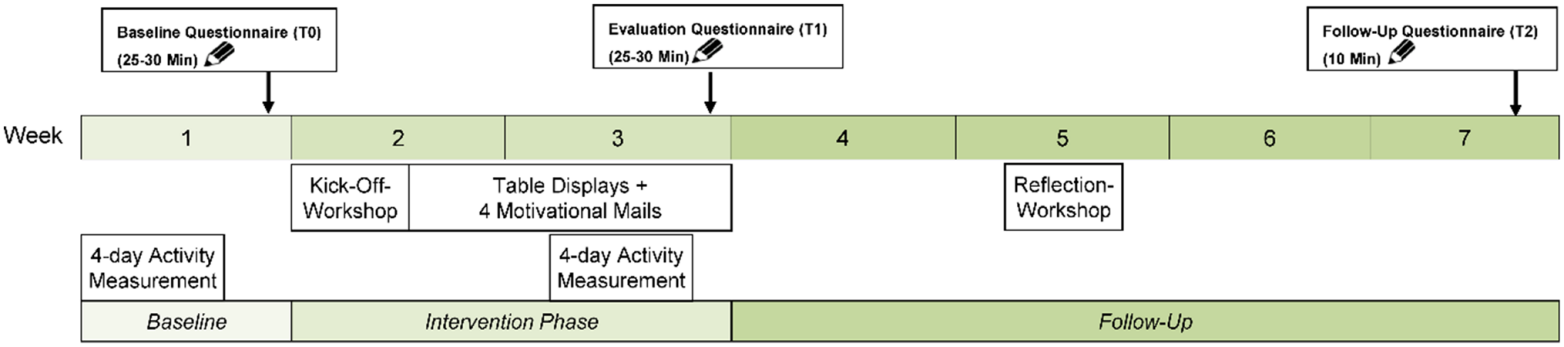
Intervention procedure

Our single arm pre-post-follow-up design consisted of an initial four-day objective measurement period of sedentary behavior that was immediately followed by a baseline questionnaire on the fifth day (T0). The *Leicht Bewegt* intervention (see below) was implemented in week 2 and 3. In week 3 also the second four-day objective measurement of sedentary behavior was conducted, immediately followed by the evaluation questionnaire (T1). The reflection workshop was organized in week 5 and in week 7 participants were asked to complete the follow-up questionnaire (T2). The intervention procedure is shown in Fig. 2.

### Intervention Leicht Bewegt

Every participating group designated a voluntary *active champion* who was coached in an one-hour online training by a member of the study team to represent the group’s main organizer and motivator during the study period. The intervention started with a kick-off workshop guided by the active champion. The workshop lasted approximately 60 min and conveyed information on sedentary behavior and associated risks. In addition, group-specific action strategies for reducing sedentary behavior during working hours were elaborated and three of them determined in a voting process. The strategies could relate to the physical environment (e.g., moving the coffee machine to a more distant location), organizational processes (e.g., introducing meetings with standing as default) and/or individual behaviors (e.g., setting movement reminders on mobile phones; see Online Resource 1 for an overview of all elaborated strategies). Participants were also handed out table displays that included 50 pages of tips, reasons, and helpful links for sitting less and moving more at work as well as blank pages for personal notes. Four motivational emails were sent to participants during the two weeks of intervention. Those contained a picture of people or things that symbolize a dynamic lifestyle together with an attention-grabbing question or sentence (for example: “That little bit of standing up doesn't help, does it?!”) and a short explanatory text to go with it (see Online Resource 2 for an example).

The reflection workshop one week after the initial intervention, again led by the active champion, lasted approximately 20 min and was intended to review the implementation phase. In this regard, feasible and effective strategies to reduce sedentary behavior were strengthened and ineffective ones were optimized to implement less sedentary working habits in the long term.

### Pretest

To check the planned procedure and surveys regarding feasibility and comprehensibility, we conducted a small pretest of the intervention with 11 participants of one DKFZ-department prior to the regular project. This led to small adaptations regarding item formulations and the intervention procedure.

### Measures

#### HAPA variables

Unless otherwise specified, items and scales were derived from the HAPA (Schwarzer 2007), which was applied in several studies (Schwarzer 2008), and partly adapted to the target behavior and context of this study.

*HAPA stage detection* To capture the current HAPA stage of the participants, namely pre-intender, intender, or actor, the participants were asked whether they consciously reduced sitting time lately. For the baseline questionnaire, the response options were “No, and I don't plan to” (pre-intender), “No, but I am thinking about it”, (pre-intender) “No, but I have the firm intention to do so” (intender), “Yes, but only recently” (actor), and “Yes, for a long time already” (actor). In the evaluation questionnaire, the last two response options were changed into “Yes, but only as a result of the *Leicht Bewegt* program” and “Yes, even before the *Leicht Bewegt* program” to identify a potential stage change as an intervention effect.

*Intention* Intention was measured with three items asking for the intention (a) to sit a smaller proportion of the workday in the upcoming weeks, (b) to regularly interrupt sitting phases during work in the upcoming weeks and (c) to integrate more activity into their daily work routine in the upcoming weeks. These had to be answered on a seven-point Likert scale from 0 (“don’t agree at all”) to 6 (“fully agree”). An additional item asked for the subjective probability to turn their intentions into action in the upcoming weeks on a percentage scale from 0 to 100%. A mean score was calculated by multiplying the fourth item by six, dividing it by 100 and then summing all four items and dividing them by four (Sieverding et al. 2010). Cronbach’s *α* was 0.78 (T0) and 0.65 (T1).

*Task self-efficacy* Task self-efficacy was assessed with three items that asked to rate how confident participants were in their ability to implement above mentioned actions that were queried for the assessment of intention (e.g., “I am sure that I can interrupt sitting phases during work after 30 min at the latest.“). Response options ranged on a four-point Likert scale from “clearly disagree” (= 0) to “clearly agree” (= 3). Cronbach’s *α* was 0.57 (T0) and 0.67 (T1).

*Risk perception* Risk perception was assessed with three items asking for risks from prolonged sitting with regard to (a) becoming chronically ill, (b) to suffer from acute or chronic pain, or (c) to develop cardiovascular disease. Each risk had to be rated on a five-point Likert scale from “much below average (= 0)” to “much above average (= 4)”. Cronbach’s *α* was 0.84 (T0) and 0.88 (T1).

*Outcome expectancies* Participants were asked to specify whether they expect positive outcomes (i.e., having more energy, feeling more at ease, and suffering from less back pain) or negative outcomes (i.e., completing fewer tasks, losing focus on work, and losing time) to happen when significantly reducing and frequently interrupting sitting phases. Answers had to be given on a four-point Likert scale from “clearly disagree” (= 0) to “clearly agree” (= 3). Cronbach’s *α* was 0.64 (T0) and 0.56 (T1).

*Maintenance self-efficacy* To measure maintenance self-efficacy, we asked the participants to rate their confidence to deal with six possible barriers for long-term reduction of sedentary behavior during work (i.e., lack of visible changes, people in the work environment who are indifferent to sitting times, a long period of habituation, situations that trigger old sitting habits, a stronger desire to sit, and a high degree of effort to change habits). Response options ranged on a four-point Likert scale from “clearly disagree” (= 0) to “clearly agree” (= 3). Maintenance self-efficacy was not included in the baseline questionnaire. Cronbach’s *α* was 0.86.

*Action/coping planning* To measure action planning, participants were asked whether they planned in the last two weeks how, when, how often, and with which strategies to reduce sitting times. For coping planning, the participants were questioned whether they had planned how to continue to reduce sitting times during the last two weeks despite feeling restricted in terms of health, feeling tired or listless, having an unusually high amount of job tasks, or failing in reducing sitting times for a few days. Response options ranged again on a four-point Likert scale from “clearly disagree” (= 0) to “clearly agree” (= 3). Action/coping planning was not included in the baseline questionnaire. Their Cronbach’s *α* was 0.74 and 0.86, respectively.

#### Activity behavior

*Subjective measurement* To assess participants’ subjective perception of their sedentary behavior during work, participants were asked to indicate how periods of sitting, standing, and walking were distributed as a percentage of a typical workday over the previous seven days. In the follow-up questionnaire, the previous three weeks were considered. In addition, participants were asked to indicate how often they interrupt their sitting time within one hour of a typical workday (from 0 to 5 times or more).

*Objective measurement* For objective measures of sedentary behavior, *activPAL 3* (Pal Technologies Ltd., Glasgow)—inclinometers with high validity (O'Brien et al. 2022)—were used. ActivPals are continuously attached to the center of the front of the thigh using the transparent and hypoallergenic 3 M Tegaderm film patch. They register the inclination of the thigh and can distinguish between sitting/lying, standing, walking, sit-to-stand and stand-to-sit transitions and step counts (Aminian and Hinckson 2012). During the phases of objective measurement (see procedure), participants were instructed to wear it during four consecutive days. The results of the measurements were not visible to the participants. After the measurement phases, recorded files were downloaded and analyzed using the device-specific software *PALanalysis*.

For each measurement day, the sum of the proportion of working hours spent walking, standing, sitting, and interruptions of sitting times were calculated. To exclude non-working hours, individual working hours were previously requested in the baseline questionnaire and all tracker records outside working hours were ignored in the analysis. In addition, the first measurement day in both measurement periods was excluded from the analysis to minimize a bias of possible habituation effects due to the unfamiliar tracker wearing.

*Satisfaction rating* To capture participants’ experiences and opinions regarding the intervention, they were asked whether they had changed their sitting behavior, whether they would like to implement the elaborated strategies in their everyday work in the future, and whether they would recommend future participation in the intervention to others. Response options were "no,” “rather no,” “rather yes,” and “yes.”

#### Background characteristics

Other measures included participants’ age (range of 18–29 years, 30–45 years, or 46–65 years), sex, their work circumstances (i.e., part-time, full-time, or freelance as well as working from home, in presence in their office, or alternating between those two options), and their general health based on the *Short-Form-Health-Survey-12* (SF-12; Ware et al. 1996).

#### Data analysis

We performed further data processing and analysis via the statistical software IBM SPSS Statistics 27. A *p* < 0.05 was considered as statistically significant. Descriptive statistics were used to describe sociodemographic characteristics of the study population, their activity behavior and their satisfaction with the intervention. To check the significance in changes of subjective (T0 to T1; T0 to T2) and objective activity measures (T0 to T1) as well as in motivational HAPA variables (T0 to T1), we used paired *t* -tests. We calculated Cohen’s *d* (Cohen 1988) to indicate effect sizes for *t* -tests (small effect: *d* ≥ 0.2, medium: *d* ≥ 0.5, and large: *d* ≥ 0.8). Pearson correlation coefficients were used to analyze bivariate correlations between motivational HAPA variables (T1) and intention (T1) as well as between volitional HAPA variables (T1) and parameters of subjective activity (T2). In order to estimate the required sample size for the planned pre-to-post comparisons in our within-subjects design, we used G*Power (Faul et al. 2009) for a power analysis. Assuming a 5% *α*-level and a power of 90%, the required minimum sample size was *n* = 36 to detect at least medium-sized effects (Cohen’s *d* = 0.50) –which is based on findings of the effect on sitting times of the Australian predecessor version of our *Leicht Bewegt* intervention (Healy et al. 2018).

## Results

A detailed description of the sample characteristics is displayed in Table 1.

**Table 1:** Baseline characteristics of the total sample

Baseline characteristic	*n*	%	*M*	*SD*
*Sex*				
Female	46	86.6		
Male	9	16.4		
*Age category*^a^				
18–29 years	8	14.8		
30–45 years	21	38.9		
46–65 years	25	46.3		
*Working hours*^b^				
Full-time	33	60.0		
Part-time	22	40.0		
*Location of work*				
Only home office	14	28.9		
Only office	6	11.1		
Alternating	33	60.0		
Subjective health^c^	45		2.42	.87

^a^*n* = 54

^b^*n* = 53

^c^Response range 0–4, higher scores indicating better health

### Adherence

Of the 55 registered participants, 52 (94.5%) participants fully completed the baseline questionnaire and 46 (83.6%) participants took part in the entire evaluation questionnaire. Participants who did not report data on primary outcomes (subjective or objective sitting behavior, or intention) at either T0 or T1 were excluded from the analyses (*n* = 10), leading to a total *N* of 45 participants. The follow-up questionnaire was completed by 38 participants. This corresponds to a dropout rate of 18.2% (T1) and 30.9% (T2).

Dropout analyses did not reveal systematic patterns nor significant differences between participants with complete and incomplete data with regard to age category, sex, health status, working hours, and work location. Due to a technical failure during the first objective measurement period, the activity behavior of the 16 first cohort participants was not recorded. Additionally, there were eight participants who could not participate in at least one of the objective measurement periods because of unavoidable absences. In total, 31 (56.4%) complete datasets with objective data from the first and second measurement period were included for corresponding analyses. There were no significant differences in subjective activity between those participants that provided objective activity data and those who did not.

During the intervention, 51 participants attended the kick-off workshops and 50 participants attended the reflection workshops of the respective groups.

### Activity behavior

**Fig. 3:**
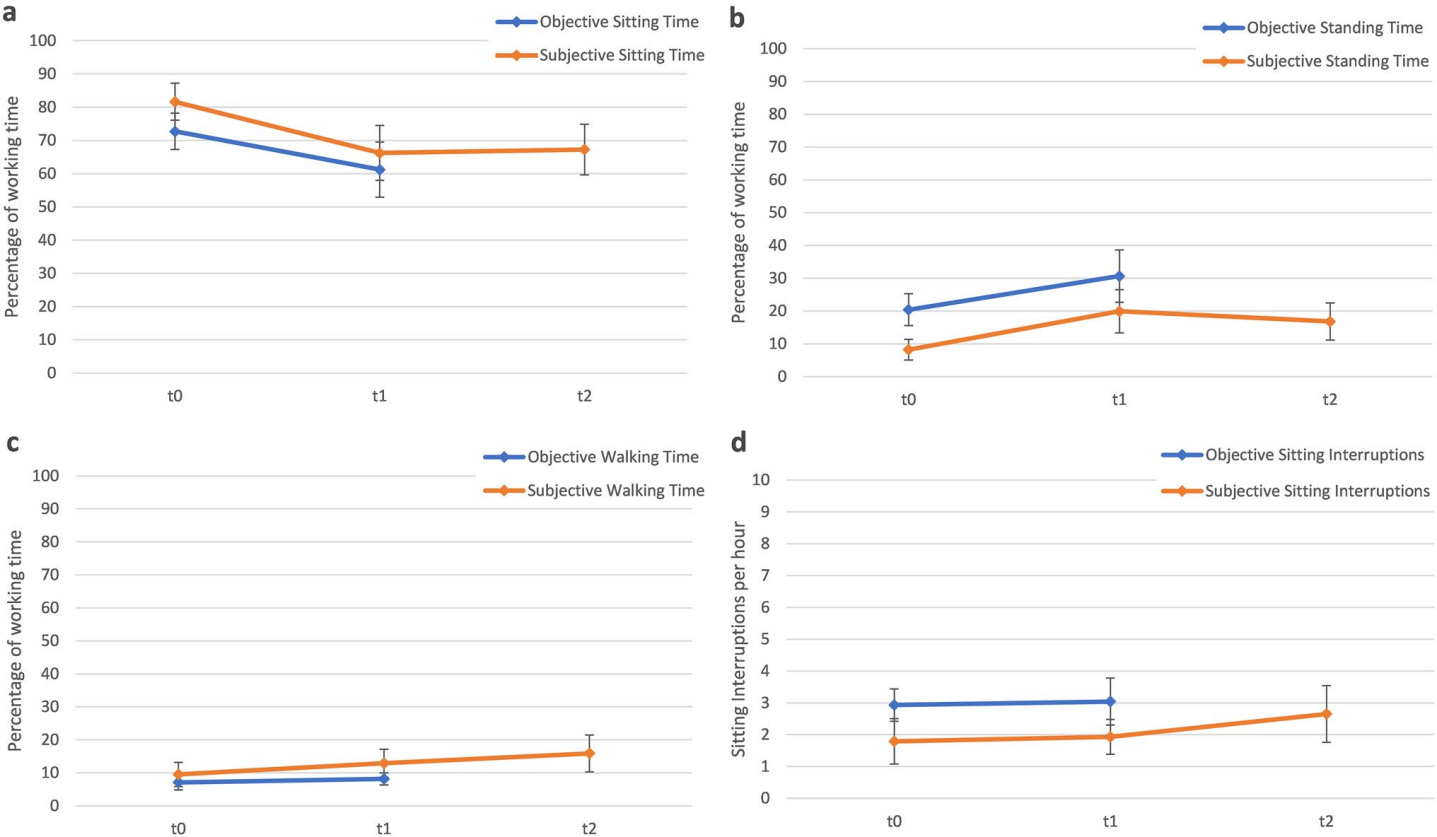
Subjective and objective activity behavior at T0, T1 and T2

Figure 3 shows the means and standard deviations of the subjective and objective measures of activity behavior at T0, T1, and T2 (exact values are given in Online Resource 3).

*Subjective measures* According to the participants’ subjective assessments, there was a significant reduction in sitting time from T0 to T1 (*p* < 0.001;* d* = − 1.14) and from to T0 to T2 (*p* < 0.001;* d* = − 1.08). Based on a working time of eight hours, this corresponds to an average decrease of 74 min (T1) or 70 min (T2) of sedentary time per workday. Subjective frequencies of sitting interruptions per hour did not increase significantly from T0 to T1 (*p* = 0.583; *d* = 0.08), but there was a trend for a significant increase from T0 to T2 (*p* = 0.055;* d* = 0.33). The subjective proportion of standing at work increased significantly from T0 to T1 (*p* < 0.001, *d* = 1.01) and from T0 to T2 (*p* < 0.001; *d* = 0.96). Furthermore, the subjective percentage of walking during work increased significantly from T0 to T1 (*p* = 0.008; *d* = 0.43) and from T0 to T2 (*p*  < 0.001; *d* = 0.64).

*Objective measures* According to the objective activity data, participants reduced their sitting time during work significantly from T0 to T1 (*p* = 0.001, *d* = − 0.66) that corresponds to an average decrease of 55 min of sedentary time per workday. The objective frequency of sitting interruptions per hour did not increase from T0 to T1 (*p* = 0.602; *d* = 0.10). The objective proportion of standing at work increased significantly from T0 to T1 (*p* = 0.002, *d* = 0.61) and, there was a trend for a significant increase of walking during work (*p* = 0.083, *d* = 0.32).

### HAPA variables

*HAPA stage development* At baseline, the sample was divided exactly in thirds (33.3%) into pre-intenders, intenders, and actors, corresponding to 15 participants per stage. This distribution clearly changed after the intervention. At T1, only one participant (2.2%) remained in the pre-intender stage. Three participants (6.7%) indicated that they had the intention to reduce sitting time but had not yet implemented it. A clear majority of 41 participants (91.9%) claimed to have consciously reduced sitting time. Thirty-one (68.9%) participants stated that this behavior change was induced by the *Leicht Bewegt* intervention.

*Development and correlations of motivational variables* Mean scores and standard deviations for each motivational variable and both measurement points are presented in Table 2. Task self-efficacy, outcome expectancies, and risk perception did not change significantly, whereas the mean intention score increased significantly from T0 to T1(*t* (44) = − 3.53, *p* = 0.001, *d* = − 0.53).

**Table 2:** Motivational variables of the health action process approach at T0 and T1

Variable	*n*	Baseline (T0)	Evaluation (T1)	*t*(44)^a^	*p*
		*M (SD)*	*M (SD)*		
Intention^b^	45	4.42 (1.04)	4.87 (.76)	− 3.53	.001
Task self-efficacy^c^	45	2.24 (.57)	2.30 (.54)	− .76	.451
Risk perception^d^	45	1.79 (.80)	1.76 (.77)	.47	.638
Outcome expectancies^c^	45	2.08 (.46)	2.09 (.44)	− .13	.898
Maintenance self-efficacy^c^	45		1.90 (.61)		
Action/coping planning^c^	44		1.63 (.63)		

^a^Paired, two-sided *t* -tests for dependent samples

^b^From 0 to 6, higher score indicating stronger intention

^c^From 0 to 3, higher scores indicating higher agreement

^d^Individual risk perception assessments on five-point Likert scales from 0 (“much below average”) to 4 (“much above average”)

With respect to associations between the motivational HAPA variables at T1, task self-efficacy correlated significantly with intention (*r* = 0.62, *p* < 0.001), and there was a trend for a positive correlation with outcome expectancies (*r* = 0.27, *p* = 0.069). The association between risk perception and intention was not statistically significant (*r* = − 0.06; *p* = 0.700).

*Correlations between volitional HAPA variables and sedentary behavior change* Table 3 shows the correlations of the volitional HAPA variables at T1 with the subjective activity behavior at T2. Only coping planning correlated significantly with one of the activity variables, i.e., with the subjective frequency of sitting interruptions, *r* = 0.53, *p* = 0.002.

**Table 3:** Bivariate correlations between volitional variables of the health action process approach and activity behavior

Variable	1	2	3	4	5	6	7
1. Sitting (T2)^a^		− .67**	− .68**	− .10	− .08	.16	− .19
2. Standing (T2)^a^			− .06	− .05	.14	− .15	− .03
3. Walking (T2)^a^				.30	− .02	− .06	.33
4. Sitting interruptions (T2)^a^					.19	.12	.53**
5. Maintenance self-efficacy (T1)^b^						.03	.31*
6. Action planning (T1)^c^							.38**
7. Coping planning (T1)^c^							

Bivariate Pearson correlations (two-sided)

*p < .05, **p < .01

^a^Self-reported

^b^Higher scores indicate more pronounced maintenance self-efficacy

^c^Higher scores indicate more detailed action/coping planning

### Satisfaction rating

The clear majority of participants (34 of 38; 89.5%) answered "yes" or " rather yes" to the question of whether their physical activity behavior at work had changed as a result of the intervention. Even more participants answered "yes" or "rather yes" to the question of whether they would like to implement the used strategies in their everyday work in the future (34 of 36; 94.4%) as well as to the question of whether they would recommend the program to their colleagues (35 of 37; 94.6%).

## Discussion

In the present study, we observed a successful reduction in the proportion of subjectively reported and objectively measured sitting during working time after the *Leicht Bewegt* intervention. Subjectively reported sitting behavior also showed a stable effect three weeks after the intervention. Correspondingly, the proportion of standing (subjective and objective) and walking times (subjective) increased as a result of the intervention. With regard to the HAPA variables, intention to reduce sedentary behavior increased from pre- to post-intervention and action planning was significantly associated with sitting interruptions at follow-up. The *Leicht Bewegt* intervention was very well accepted and evaluated by the study participants and could be an easy-to-implement tool to reduce sedentary behavior in other work environments as well.

The reduction in the sitting time achieved by the intervention means a practically relevant change in activity during working hours. With an objectively measured decrease in the sitting time of 11.5%, a full-time worker sat on average 55 min less per workday. The subjectively perceived reduction in sedentary time, based on a higher number of participants, amounted to even 15.4% corresponding to a decrease of 74 min per workday. This reduction is in the upper range of effects of interventions on sedentary behavior in the adult workplace found in an umbrella review (15–77 min per (work)day) (Nguyen et al. 2020) and 25 min higher than in a comparable study of the intervention’s Australian predecessor version BeUpstanding (Healy et al. 2018). The effect found in this study is encouraging but should be interpreted with caution given the small sample size. Nevertheless, the objective data were consistent with the trend of subjectively perceived change of sitting behavior, suggesting an actual intervention effect. The high correspondence between objective and subjective activity data in our study is rather unusual as people tend to have difficulty subjectively reporting their actual sitting time (Atkin et al. 2012). Sitting is in most cases an unconscious habitual behavior that is of little personal relevance (Gardner 2015; Vallacher and Wegner 1987). However, it can be assumed that our study successfully encouraged participants to be more aware of their sedentary behavior.

Our findings indicated an increase of standing time as a result of the reduced sitting time but provided an inconclusive picture with regard to the proportion of walking and the frequency of sedentary breaks. Thus, the present study suggests that most of the avoided sitting is replaced by standing. This is in line with previous workplace interventions that found higher effects on standing time (28.5 min to 127.0 min/workday) than on walking time (1.8 min to 14.0 min/workday) (Chau et al. 2014; Graves et al. 2015; Healy et al. 2013; Neuhaus et al. 2014a, b; Rollo and Prapavessis 2020a). Office workers, in particular, like the participants in our study, are usually tied to a permanent, seated work location, limiting opportunities to change work locations or to work while walking. In this regard, active workplaces that allow walking or cycling while working offer a promising way to replace sitting time not only with standing but also with (light) physical activity (Torbeyns et al. 2014).

This study also aimed to identify possible mechanisms of the intervention via changes in psycho-cognitive variables based on the HAPA model. The intention to initiate a healthier activity behavior increased significantly from pre to post-intervention. The effect matched with the results of the HAPA stage item that indicated a clear increase of intenders. Most of these new intenders attributed their intention to the intervention. Hence, the increase in intention can be interpreted as a very probable intervention effect. In contrast, task self-efficacy, outcome expectations, and risk perception did not increase significantly from pre-to post-intervention. This result pattern is in line with a study by Rollo and Prapavessis (2020a) which found a significant main effect of a sedentary behavior intervention on intention. As in our study, their intervention also had no significant effect on risk perception and self-efficacy. Deviating from our findings, they found a significant effect on outcome expectancies. In our study, participants’ mean values for self-efficacy as well as for outcome expectations were on average located between the two highest response options “partially agree” and “clearly agree” even before the intervention began. Thus, there was limited scope for significant increases. The non-significant change in risk perception may be due to item content as derived from Schwarzer (2007), which does not refer to specific consequences of sedentary behavior but to general health risk. To explore individual risk perception more precisely in the relationship between sedentary behavior and health risks, future studies may refer specifically to adverse consequences of sedentary behavior. In addition, explicitly mentioning risks of sedentary behavior during the intervention may increase risk perception. However, if participants assumed the intervention’s effect of less sedentary behavior to be persistent, they would logically have to assume a lower health risk for themselves.

The intention to reduce sedentary behavior correlated significantly with task self-efficacy at T1. No significant correlation was found between the outcome expectancies and risk perception with intention. This is in line with findings by Schwarzer (2008) who underlined the particularly important role of task self-efficacy. He describes outcome expectancies and risk perception as a starting point to contemplate about an intention formation and task self-efficacy as the variable that more directly leads to the development of an intention.

With regard to the volitional variables of the HAPA model, there was a significant association between coping planning and sitting breaks. Coping planning was identified as an effective tool to foster behavior change (Kwasnicka et al. 2013) and may be particularly beneficial to reduce sedentary behavior. A correlation between coping planning and sitting-related variables had previously also been found in an intervention on office workers (Rollo and Prapavessis 2020a) and university students (Dillon et al. 2022). In a structured context such as a workday in the office, barriers might be particularly predictable and thus remediable through appropriate planning (Gardner et al. 2016). This could also explain the success of past planning-based interventions to reduce sedentary behavior (Dillon et al. 2022; Rollo and Prapavessis 2020a; Sui and Prapavessis 2018).

Other volitional variables showed no significant correlations with sitting behavior in this study. This might be due to the nature of the intervention that allowed participating groups to choose strategies to reduce sitting time that suited their work reality and did not explicitly target individual behavior planning. Other intervention concepts that involve individual goal planning with personal counseling (Adams et al. 2013) may achieve stronger effects of volitional variables on sitting time.

A clear majority of participants was satisfied with the intervention process, motivated to implement strategies in their future work-life and would recommend the intervention to colleagues. This indicates that the intervention was perceived as pleasant and suitable for the work. Occupational interventions should ideally have a good fit to the workplace to achieve the best possible results (Backé et al. 2018). It must be mentioned, however, that questions on satisfaction with the intervention were completed only by those who participated to the end. In this respect, negative experiences could be missing here because the respective participants had already dropped out earlier. Nevertheless, adherence to the intervention was satisfactory. In medication treatment studies, good adherence is typically defined as taking at least 80% of the prescribed doses (Burkhart and Sabaté, 2003); an equivalent rate could be achieved in the *Leicht Bewegt* intervention. Nevertheless, it might be helpful for the future development of the intervention to consider how adherence could be improved, e.g., through a closer guidance of study participants.

### Strengths and limitations

This study presents an effective intervention on sedentary behavior that can be easily integrated into daily work routines. Sedentary behavior was measured objectively with validated devices promising accurate and trustworthy results regarding sitting duration (Sellers et al. 2016). Thus, the study circumvents a central weakness—the solely subjective measurement—of other intervention studies in the area of sedentary behavior. Furthermore, potential effects and mechanisms of the intervention were examined based on a variety of motivational and volitional variables of the established HAPA model. This provides valuable insights for the further development of the *Leicht Bewegt* intervention and similar approaches. However, results of the study must be interpreted considering some limitations. First, a limiting factor for the generalizability of the study results is the Covid-19 pandemic situation during the data collection which may explain why the majority of participants indicated working from home at least partially. A recent study showed that working at home office is associated with high sedentary time and linked to different environmental and motivational determinants (Niven et al. 2023). Therefore, study results may not be (fully) applicable to on-site work. Second, the proportional lack of men among the participants further limits the generalizability of our findings. Future studies should specifically try to recruit more men who showed higher sitting times than women in Germany (Froböse and Wallmann-Sperlich 2023). This would allow for analysis of sex differences in sedentary behavior and with regard to intervention effects. Third, the pre-post design involves the central disadvantage that changes in any variable cannot be attributed clearly to an intervention effect. To be able to draw causal conclusions, a randomized experimental design with a control group is needed. However, our study was designed as a pilot aiming for first insights on feasibility and effectiveness. As a fourth limitation, we did not systematically examine the reasons for dropout, which makes it difficult to improve adherence to future interventions based on the *Leicht Bewegt* program. Fifth, available data for objective sedentary behavior were reduced due to technical failure during the first activPAL measurement. Notably, there were no significant differences in subjective activity data between those for whom objective activity data were available and those for whom they were not, so selection bias due to this loss of data is unlikely. Concluding, despite the small sample size that usually increases the risk of type II errors (Columb and Atkinson 2016), we found significant effects with moderate to high effect sizes. Albeit no causality may be inferred from this, those findings suggest a meaningful intervention effect.

## Implications and conclusion

The most common obstacle to implement workplace health promotion is the lack of willingness to provide the time and financial resources (Bechmann et al. 2011; Quirk et al. 2018). Therefore, interventions should limit its costs and scope to the lowest extent needed. With its flexible toolkit approach, the *Leicht Bewegt* intervention already pursued steps in this direction. Future research should further elaborate on the effectiveness of specific intervention components to give more precise recommendations. A key question here is under which conditions a single intensive phase of an intervention is sufficient for a sustainable sedentary time reduction, e.g. the role of supervisor support or acceptance at the management level. For example, correlative findings indicated that the more individuals perceived their supervisor as supportive of active breaks, the more likely they were to experience less sedentary time (Lafrenz et al. 2018). Furthermore, one could compare the intervention components of *Leicht Beweg*t to components of other interventions such as the *Stand More AT (SMArT) Work intervention* or *SMART Working and Life (SWAL) intervention* that appear to be effective in the long term (Edwardson et al. 2018, 2022) but require more financial resources (Cox et al. 2022).

The continued effects of the intervention at follow-up suggests that its effect on the activity behavior of participants at work is sustainable, at least in a short time frame. However, for assuming “maintenance” of a desired behavior, it needs to persist for at least six months (Prochaska and DiClemente 1982). Therefore, longer assessment periods are needed to capture long-term behavior change. The present study provided an inconclusive picture regarding a potential increase of light movement as an intervention effect. There are certain limits to such an increase depending on the type of work. Nevertheless, future studies should examine whether a stronger focus on light movement would promote an even healthier replacement of sedentary time. For example, in *Leicht Bewegt*, it would be imaginable to determine that at least one group strategy not only leads to the avoidance of sitting, but also explicitly to an increase of in light movement. In addition, office workers with different focuses (e.g., administrative or leadership) have different work routines and presumably different opportunities to reduce sedentary behavior. Future studies should examine workplace roles in more detail and analyze whether they mitigate the effectiveness of strategies to reduce sedentary behavior. This may contribute to providing office workers with individualized behavioral changes techniques to reduce their sedentary time in the future.

To conclude, the present study provides preliminary evidence for the *Leicht Bewegt* intervention’s feasibility and effectiveness to reduce sitting times during office work. With a reduction of sedentary behavior of 55 min (objective measurement) or 74 min (subjective indication), it exceeded the effect of most other comparable interventions. Furthermore, the intervention offers a useful and easy-to-integrate concept that may be applied to other work sites for promoting a more active workday. To gain more clarity about long-term and comparative effectiveness, longer study periods, more rigorous designs, and a comparison with different intervention approaches should be considered.

### Supplementary Information

Below is the link to the electronic supplementary material.

Supplementary file1 (DOCX 961 KB)

## Data Availability

The authors declare that they have full control of all primary data and that they agree to allow the journal to review our data.
