# Supplementary material for: What it takes to reduce sitting at work: a pilot study on the effectiveness and correlates of a multicomponent intervention
Source: Int Arch Occup Environ Health. 2023 Nov 10;97(1):9–21. doi: 10.1007/s00420-023-02020-4 (PMC10791766; doi:10.1007/s00420-023-02020-4)
Supplement: Supplementary file 1 — Supplementary file1 (DOCX 961 KB) [file 420_2023_2020_MOESM1_ESM.docx]

**Supplementary Information (SI)**

**Title:** What it Takes to Reduce Sitting at Work: A Pilot Study on Factors of Sedentary Behavior Change Within a Multicomponent Intervention

**Journal name:** International Archives of Occupational and Environmental Health

**Authors:** Jannik Porath, Laura I. Schmidt, Juliane Möckel, Chiara Dold, Lisa Hennerkes, Alexander Haussmann

**Corresponding Author:** Alexander Haussmann, German Cancer Research Center and National Center for Tumor Diseases Heidelberg and University of Heidelberg, alexander.haussmann@nct-heidelberg.de

| **Online Resource 1.** *Action strategies of the groups to reduce sitting time.* | |
| --- | --- |
| action strategy | number of groups mentioning this strategy, *k* |
| - Setting movement/posture reminders on private devices like phone or smartwatch | 4 |
| - Introducing meetings with standing as default or at least as explicitly communicated option | 3 |
| - Integrating a walk or another movement part into the break | 3 |
| - Making deliberate detours on the walks within the workday | 2 |
| - Use of the stairs whenever possible | 2 |
| - Moving the fridge and coffee machine to a more distant location. | 1 |
| - Make phone calls while standing or walking | 1 |
| - Using a group chat to share tips, reminders and increase motivation | 1 |
| - Participating in “Active Break” courses of the occupational health management | 1 |

**Online Resource 2.** *Example of a motivation email.*


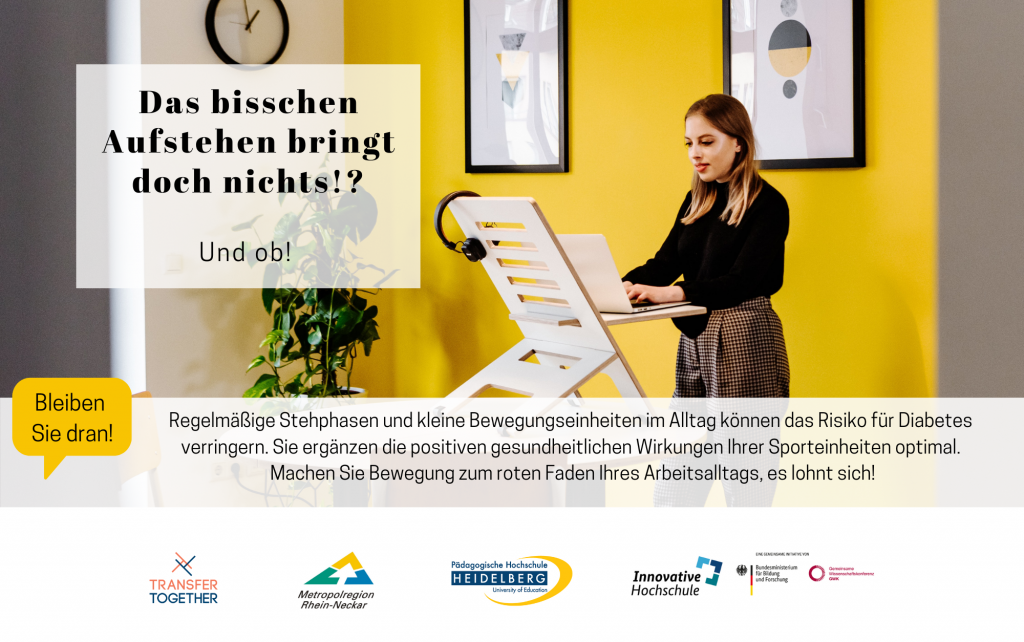


**Online Resource 3.** *Subjective and objective activity behavior change during the study.*

|  |  | Baseline (T0) | |  | Evaluation (T1) | |  | Follow-Up (T2) | |
| --- | --- | --- | --- | --- | --- | --- | --- | --- | --- |
| Activity Behavior Measure | *n* | *M* | *SD* | *n* | *M* | *SD* | *n* | *M* | *SD* |
| Subjective Sitting Time^a^ | 45 | 81.64 | 11.20 | 45 | 66.22 | 16.52 | 38 | 67.24 | 15.19 |
| Subjective Standing Time^a^ | 45 | 8.22 | 6.34 | 45 | 19.91 | 13.20 | 38 | 16.82 | 11.30 |
| Subjective Walking Time^a^ | 45 | 9.51 | 7.32 | 45 | 12.91 | 8.42 | 37 | 15.89 | 11.12 |
| Subjective Sitting Interruptions^a^ | 45 | 1.79 | 1.42 | 45 | 1.93 | 1.10 | 36 | 2.67 | 1.81 |
| Objective Sitting Time^b^ | 31 | 72.74 | 10.88 | 31 | 61.21 | 16.63 | - | - | - |
| Objective Standing Time^b^ | 31 | 20.40 | 9.67 | 31 | 30.63 | 15.92 | - | - | - |
| Objective Walking Time^b^ | 31 | 7.09 | 4.54 | 31 | 8.16 | 3.63 | - | - | - |
| Objective Sitting Interruptions^b^ | 31 | 2.93 | 1.01 | 31 | 3.04 | 1.48 | - | - | - |

*Note: M* = mean*; SD* = standard deviation

^a^ Subjective assessment of the respective behavior’s percentage of a typical workday

^b^ Objectively measured percentages of the respective behavior while working
